# Evolutionary, structural and functional insights in nuclear organisation and nucleocytoplasmic transport in trypanosomes

**DOI:** 10.1002/1873-3468.14747

**Published:** 2023-10-15

**Authors:** Norma E. Padilla‐Mejia, Mark C. Field

**Affiliations:** ^1^ School of Life Sciences University of Dundee UK; ^2^ Institute of Parasitology, Biology Centre Czech Academy of Sciences České Budějovice Czechia

**Keywords:** evolutionary diversity, nuclear lamina, nuclear pore complex, nucleus, trypanosoma

## Abstract

One of the remarkable features of eukaryotes is the nucleus, delimited by the nuclear envelope (NE), a complex structure and home to the nuclear lamina and nuclear pore complex (NPC). For decades, these structures were believed to be mainly architectural elements and, in the case of the NPC, simply facilitating nucleocytoplasmic trafficking. More recently, the critical roles of the lamina, NPC and other NE constituents in genome organisation, maintaining chromosomal domains and regulating gene expression have been recognised. Importantly, mutations in genes encoding lamina and NPC components lead to pathogenesis in humans, while pathogenic protozoa disrupt the progression of normal development and expression of pathogenesis‐related genes. Here, we review features of the lamina and NPC across eukaryotes and discuss how these elements are structured in trypanosomes, protozoa of high medical and veterinary importance, highlighting lineage‐specific and conserved aspects of nuclear organisation.

## Graphical abstract

We consider the features of the nuclear lamina and NPC by comparing humans, yeasts and trypanosomes. We discuss how those nuclear elements are structured in trypanosomes and how they differ from, or are conserved with other eukaryotic lineages. We also discuss the functional and evolutionary aspects of those fundamental elements of nuclear structure.

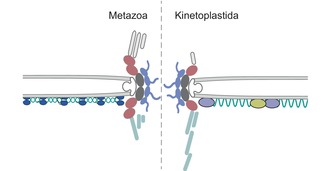

## Abbreviations

**BES**, bloodstream expression site

**BSF**, bloodstream form

**CF**, cytoplasmic filaments

**Cr**, *Chlamydomonas reinhardtii*

**CRWN**, crowded nuclei protein

**Dbp5**, RNA helicase DEAD box protein 5

**Esc1**, establishes silent chromatin 1 (protein)

**FG‐Nup**, FG‐repeats nucleoporin

**FG‐repeat**, phenylalanine‐glycine repeat

**Gle1/2**, mRNA export factor Gle1 or Gle2

**Gp210**, nuclear pore membrane glycoprotein 210

**Hs**, *Homo sapiens*

**HAT**, human African trypanosomiasis

**IR**, inner ring

**LAD**, lamin‐associated domains

**LECA**, last eukaryotic common ancestor

**Mex67**, mRNA export factor 67

**Mlp1/2**, myosin‐like protein 1 or 2

**MR**, *trans*‐membrane scaffolding ring

**mRNA**, messenger RNA

**mRNP**, messenger ribonucleoprotein

**Mtr2**, mRNA transport regulator

**NB**, nuclear basket

**Ndc1**, nuclear division cycle protein 1 (nucleoporin)

**NE**, nuclear envelope

**NE81**, 81 kDa nuclear envelope protein

**Nic96**, 96 kDa nucleoporin‐interacting component

**NLS**, nuclear localization signal

**NMCP**, nuclear matrix constituent protein

**NPC**, nuclear pore complex

**Nsp1**, nucleoskeletal‐like protein 1

**Nup**, nucleoporin

**NUP‐1**, nucleoporin 1

**NUP‐2**, nucleoporin 2

**Nxf1**, nuclear RNA export factor 1

**Nxt1**, NTF‐2 related export protein 1

**OR**, outer ring

**PCF**, procyclic culture form

**Pf**, *Plasmodium falciparum*

**POM**, pore membrane protein

**RBP10**, RNA binding protein 10

**Sc**, *Saccharomyces cerevisiae*

**Sec13**, transport protein Sec13

**Seh1**, nucleoporin Seh1 (Sec13 homologue 1)

**Tb**, *Trypanosoma brucei*

**Tg**, *Toxoplasma gondii*

**Tpr**, translocated promoter region protein

**TREX1/2**, three‐prime repair exonuclease 1 or 2

**tRNA**, transfer RNA

**TY1**, epitope tag TY1

**VSG**, variant surface glycoprotein.

Protozoan parasites cause a plethora of human, animal and plant diseases, many of which are fatal without intervention and have profound impact on economic activity and quality of life. The class Kinetoplastida (clade Discoba) [1] are flagellated protozoa possessing a kinetoplast, an unusual configuration of mitochondrial DNA [2, 3]. The Kinetoplastida include *Trypanosoma* and *Leishmania* species, causative agents of multiple diseases, with particular importance to tropical and subtropical countries, although outbreaks in North America, Europe (especially the Mediterranean belt) and Oceania are occurring with increased frequency [4, 5]. Human African Trypanosomiasis (HAT), or sleeping sickness, is caused by *Trypanosoma brucei gambiense* and *Trypanosoma brucei rhodesiense* and is fatal if untreated [5]. Veterinary infections, with a spectrum of severity and clinical symptoms, are caused by *T. evansi*, *T. equiperdum*, *T. congolense* and *T. vivax* and are distributed across Africa, Asia and Latin America; death of cattle and domestic animals due to trypanosomiasis causes huge economic loss in these regions [4, 6]. *T. b. gambiense* and *T. b. rhodesiense* are transmitted by over 20 species of tsetse flies infected *via* blood feeding. The life cycle of *T. brucei* includes up to seven different forms developing in the mammal host and the tsetse fly [7], with the bloodstream form (BSF, mammal host) and the procyclic culture form (PCF, insect host) being important experimental models as they can be cultured *in vitro* and are amenable to cell and molecular biology techniques.

Trypanosoma species are characterised by an elongated cell body of 10–20 μm propelled by a single flagellum [8]. Differentiation in these protozoa is a complex process involving a series of physiological and biochemical changes, one of the most distinctive being the replacement of the surface coat of variant surface glycoprotein (VSG) in the BSF to procyclin in the PCF [7]. VSG is expressed at a very high level and switches between paralogous genes with high frequency to facilitate immune evasion *via* antigenic variation [9, 10]. VSG genes are transcribed from bloodstream expression sites (BESs) located at subtelomeric regions. Only a single BES is active and this monoallelic expression is central for immune evasion [11]. Moreover, one of the striking features of *T. brucei* is the unusual transcription of VSG and procyclin by RNA Polymerase I [12].

Drugs have been developed against all human trypanosome pathogens, *albeit* with differing levels of efficacy due to toxicity, drug resistance and other factors [13, 14]. When drugs are combined with efficient local healthcare, the prognosis is very good and for HAT has achieved spectacular progress; disease occurrence reached a historic prevalence of under 1000 cases in 2018, remaining below that during 2022, and declared effectively eliminated from multiple countries [15]. However, in other cases, such as the American trypanosome, *Trypanosoma cruzi*, the causative agent of Chagas disease, the impact on human health remains severe, affecting 6–8 million people in the American continent, with up to 100 million people at risk of infection [16, 17]. Another example of kinetoplastid is *Leishmania* species, the causative agents of leishmaniasis, a disease with a range of clinical manifestations and severities and present in at least 88 countries with more than 350 million people at risk [18]. These two pathogens demand ongoing campaigns for disease control including characterisation of biochemical and genetic mechanisms of pathogenesis/virulence and the identification of new drug modalities [13, 14].

The nucleus encloses genomic DNA within the nuclear envelope (NE), a double lipid bilayer associated with a filamentous meshwork or lamina residing at the inner face, providing mechanical support and contributing to multiple functions. Transport between the nucleus and cytoplasm occurs through the nuclear pore complex (NPC), a massive macromolecular gateway constructed from several hundred subunits and with involvement in regulating gene expression and other functions. In evolutionary biology, the last eukaryotic common ancestor, or LECA, is considered the first eukaryote and is minimally defined by having a mitochondrion, flagellum and a nucleus [19, 20, 21]. Additionally, the LECA also possessed a NE, NPCs and a nuclear lamina, with additional subcellular structures such as a cytoskeleton and membranous endosomal and exocytic compartments. Complex metabolism and gene regulation were also present in the LECA [19, 20, 21]. Widespread ultrastructural and genome features are likely to have been inherited from the LECA [20]. After the LECA, different lineages adapted their nuclei such that the nuclear lamina and NPC likely co‐evolved with diverging mechanisms of gene expression, transcription and RNA processing. As the LECA bore NPCs and a nuclear lamina [19], it is interesting to consider how these crucial nuclear structures have diversified across eukaryotes.

Trypanosomes likely diverged from other eukaryotes over a billion years ago [22], and this deep distinction is reflected in multiple aspects of their biology, including their own nuclear structures that possess taxa‐specific components [23]. Thus, apart from their medical relevance, they are a valuable model to gain insight into evolutionarily eukaryotic structures and processes. Here, we discuss the biology of the trypanosome nucleus, focusing on the nuclear lamina and NPC, emphasising the importance of these structures in the biology of these pathogenic protozoa. We will make comparisons *versus* the canonical models of mammalian cells and other recurrent biological models such as fungi and plants to highlight how trypanosomatid structures have evolved and diverged. Here, we take the nuclear lamina and NPC as fascinating examples of evolution and diversity across eukaryotes, but also linked to mechanisms of pathogenesis.

## The nuclear lamina

**Fig. 1:**
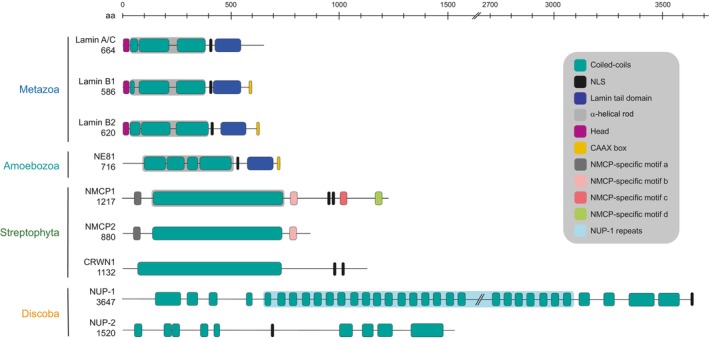
Comparison of major lamina protein architectures across eukaryotes. Main features are shown for experimentally characterised lamins; structural motifs/domains of relevance are shown on the right represented by different colours. Information taken from Uniprot [146], Trytryps database [65] and literature [49, 52, 53, 147, 148]. Number of aminoacids is indicated under each protein name. Only mature protein features are shown, pre‐lamin A/C contains a CaaX box that is lost from the mature protein. Notice that all proteins are rich in coiled‐coils domains (teal).

At the inner face of the NE the nucleus is surrounded by the nuclear lamina, a filamentous meshwork that in metazoa is constituted of coiled‐coil lamin proteins [24] (Fig. 1). The lamina is the major structural element of the nucleus, influencing nuclear shape, size and structural integrity [25]. Furthermore, lamina‐mediated mechano‐signalling allows the transduction of mechanical signals from the cytoskeleton to the nucleoplasm by direct connection of these structures through NE‐located *trans*‐membrane protein complexes. The lamina also interacts directly with the NPC and multiple NE proteins, influencing NE organisation [26].

Mammalian lamins are coiled‐coil proteins of ~ 60–80 kDa, and belong to the intermediate filament family; they are evolutionary conserved across Metazoa [27]. There are four major lamin subtypes, encoded in humans by the *LMNA*, *LMNB1*, *LMNB2* genes to express lamin A and lamin C (generated by alternative splicing), lamin B1 and lamin B2, respectively. Lamin mutations cause heritable diseases termed laminopathies with a range of cardiac, muscular–skeletal and other issues and over 600 lamina mutations are linked to pathogenesis [28, 29]. These proteins consist of a short globular N‐terminal domain (head) followed by a central α‐coiled‐coil domain (rod) and a globular C‐terminal domain containing a lamin‐tail domain incorporating an immunoglobulin fold, a nuclear localisation signal and a C‐terminal CaaX prenylation signal [30] (Fig. 1). In B‐type lamins the C‐terminus is farnesylated at the cysteine residue, which increases the affinity for membranes and is necessary for lamin B localisation and network organisation [31]. Pre‐lamin A is prenylated but the modification is cleaved and lost during maturation, while lamin C lacks the CaaX signal and is hence never prenylated [30]. Lamins form homodimers that associate with other dimers in a strict head‐to‐tail fashion to form filaments [32]. In mammalian nuclei, each lamin isoform forms a separate meshwork [33] that interconnects and interacts in a complex manner. These connections are codependent as depletions in one lamin isotype impact the equilibrium of the entire lamin meshwork and nuclear morphology [26, 30, 34, 35, 36]. Furthermore, B‐type lamins are attached to the inner nuclear membrane, while A‐type lamins face closer towards the nucleoplasm. Interestingly, in a further diversification of function, NPC components interact more closely with lamin C compared with lamin A [36].

Beyond structural roles, in mammalian cells, lamins participate in controlling genome architecture, DNA replication and transcription by interactions with chromatin (*via* lamin‐associated domains, LADs), chromatin‐modifying enzymes and transcription factors [37, 38]. Lamins A/C can shuttle to the nucleoplasm where they bind and regulate euchromatic regions and interact with transcription and cell cycle regulators [39]. In mammalian cells, lamins are phosphorylated differentially during the cell cycle, i.e., some residues are phosphorylated at the onset of mitosis with others modified during interphase and mitosis [40]. Phosphorylated lamins regulate gene expression through enhancer binding [41], expanding the roles of phosphorylated lamins during interphase. Importantly, lamins support the mechanism of cell division, as they solubilise during open mitosis to allow full breakdown of the NE in a highly regulated process that includes serine phosphorylation that induces lamin solubility and disassembly [42]. However, this is not a universal feature as in many organisms that possess lamins or lamin analogues, mitosis is closed or semi‐closed and the NE does not break down, e.g., *T. brucei* [43] and *Dictyostelium discoideum* [44, 45].

**Fig. 2:**
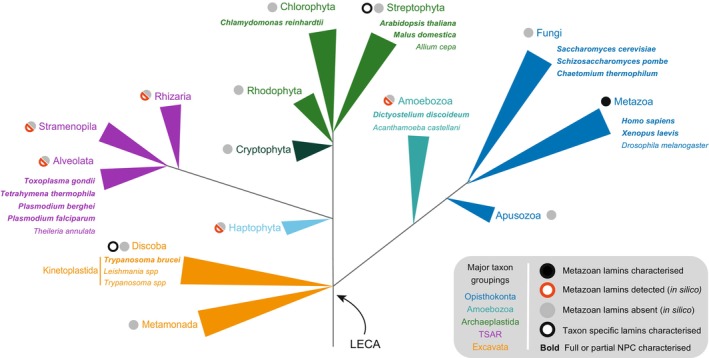
Characterisation of lamins and NPCs in eukaryotic lineages. A phylogenetic tree showing the presence or absence of lamin or lamin‐like proteins across different taxa. Major groups are indicated in the legend at the right (grey box) and representative species are included. Branches in the same colour correspond to the same super kingdom, e.g., Metazoa, Fungi and Apusozoa all correspond to Opisthokonta. LECA indicates the position of the LECA. The tree is based on Burki *et al*. [149]. Circles next to the taxa name indicates presence/absence of lamin proteins; black circle, mammalian (metazoan) lamins found; red circle, metazoan lamins detected *in silico* only without experimental characterisation; grey circle, metazoan lamins absent when using bioinformatic approaches; black empty circle, taxa‐specific proteins found exhibiting lamina properties. More of one of these descriptions can apply for certain groups, e.g., TSAR, Haptophyta and Amoebozoa include species that, by bioinformatic analysis, show presence or absence of metazoan lamins, e.g., in Amoebozoa, some species have a mammalian lamin homologue (*Dictyostelium discoideum*), while others lack those (*Acanthamoeba castellani*). Divergent taxa‐specific lamin proteins with lamina properties have been characterised in Discoba and Streptophyta, which lack canonical lamins. Presence/absence of lamins is based on published analysis [27, 46]. Names of organisms in which the NPC has been fully or partially characterised are in bold.

Lamins were initially considered Metazoa‐restricted, but lamin orthologs have been identified in Rhizaria, Alveolata, Amoebozoa and other lineages (Fig. 2) [27, 46]. Lamins are not universal and even closely related organisms in the same taxa may, or may not, have lamin genes, e.g., in Amoebozoa, the species *D. discoideum* and *Acanthamoeba castellani* have and lack a lamin ortholog, respectively [27, 46]. Some organisms lack proteins homologous to lamins but possess proteins that partially mimic lamin functions, e.g., Esc1 in *Saccharomyces cerevisiae*, a protein localising to the nuclear periphery that interacts with the NPC and has chromatin‐silencing properties [46, 47, 48]. In spite of the *in silico* identification of divergent lamin orthologs across a wide range of eukaryotes (Fig. 2), few lamin and lamina‐like systems are experimentally characterised, including NE81 in *D. discoideum* (Amoebozoa), an orthologue of lamin B [44, 45]; NMCP1/2 in plants (CRWN in *Arabidopsis thaliana*) [49, 50, 51] and NUP‐1/NUP‐2 in *T. brucei* [52, 53] (Figs 1 and 2). Therefore, defining a lamin analogue no longer resides with sequence alone but rather needs to consider structural and functional similarities, such as coiled‐coil organisation, nuclear peripheral distribution, regulation of nuclear shape and size and interaction and regulation of chromatin.

As homologues of Metazoan lamins have been found in several lineages (Amoebozoa, Rhizaria, Stramenopila, Alveolata, Haptophyta [27, 46, 54]; Fig. 2), lamins must have been present very early in eukaryotic evolution, *circa* the LECA [27, 46, 54]. It has also been suggested that lamins are polyphyletic, i.e., they had multiple origins during eukaryote evolution [27]. However, as the gene and species trees from lamins are concordant, this seems unlikely [46]. It is unknown if different lamina systems (namely mammalian lamins, plant NMCPs or trypanosoma NUP‐1/NUP‐2) originated by evolutionary divergence or convergence as while they contain a conserved coiled‐coil/α‐helical rod structure there is extreme divergence in size and sequence among them [27, 54, 55]. Importantly, the LECA can be considered a single cell or a population [56]. As a population with an available pool of genes [56], it is possible that the LECA possessed all three types of lamin proteins, lamin‐like, NMCP‐like and NUP‐1/NUP‐2‐like, but that only one was retained in different organisms [54, 55]. Alternatively, trypanosomes and plants may have replaced lamins with NUP‐1/NUP‐2 or NMCP, respectively [54, 55]. The restriction of the latter two systems to single lineages would support the replacement model.

**Fig. 3:**
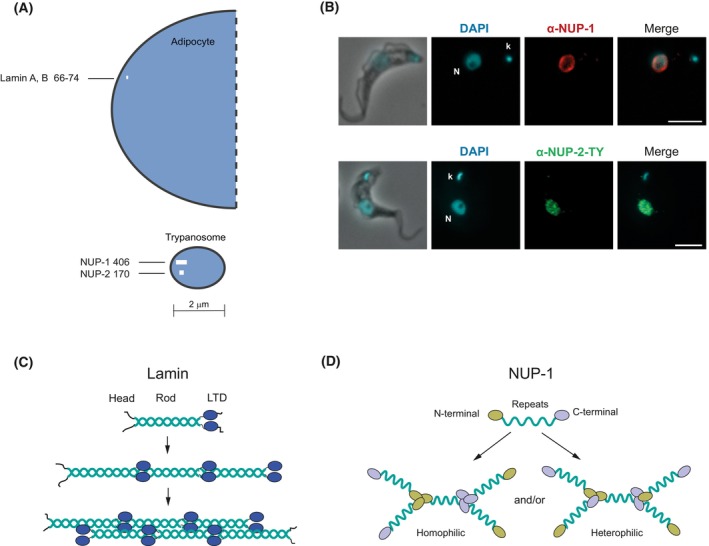
NUP‐1 features in *Trypanosoma brucei*. (A) Schematic highlighting differences in size between mammalian (adipocyte) (~ 10 μm) and trypanosome nuclei (~ 2 μm). Corresponding lamina‐constituting proteins are shown as a white line at approximate scale and molecular weights in kilodaltons. The cartoon only considers the elongated, primary structure of the proteins. Length only is significant, not the width of the line. The schematic is to emphasise that NUP‐1/NUP‐2 are huge proteins in a smaller nucleus when compared to equivalent human structures. As the sizes and sequences of the proteins are radically different, the inference is that the overall structure/assembly of the lamina in each case is likely to be distinct (see C and D). The potential volume that each lamina protein can access is clearly very different between mammals and trypanosomes. This representation is based on protein and nucleus size only and does not consider aspects such as higher‐order assembly nor secondary/tertiary structure. (B) NUP‐1 and NUP‐2 detected at the nucleus by immunofluorescence assays. *T. brucei* BSF cells showing nucleus (N) and kinetoplast (k) (the mitochondrial DNA). Cells were probed with an α‐NUP‐1 antibody (red) (top panel) or α‐NUP‐2‐TY1 (green) (bottom panel). Scale bar 5 μm. Microscopy images taken by N.E. Padilla‐Mejia. (C) Assembly of mammalian lamins. Lamins exist as a homodimer that assemble first in a parallel manner and then in an antiparallel way to form filaments. (D) Assembly of NUP‐1 molecules. NUP‐1 can form interactions with other NUP‐1 molecules in a head‐to‐head, tail‐to‐tail (homophilic interactions) or head‐to‐tail (heterophilic interactions).

In African trypanosomes, NUP‐1/NUP‐2 are lamina analogs taxonomically restricted to Kinetoplasts [46, 52, 53]. As mentioned, NUP‐1 (406 kDa) [52] and NUP‐2 (170 kDa) [53] are coiled‐coil proteins lacking lamin domains [46] such as the lamin tail domain or the CaaX motif (Fig. 1). Originally detected by subnuclear fractionation and immuno‐electron microscopy at the nucleoplasmic face of the NE [57], NUP‐1 has an N‐terminal region followed by a central rod containing a region of 17 repeats of 144 amino acids and finally a C‐terminal domain bearing an NLS [52] (Fig. 1). NUP‐2 possesses a central monopartite NLS and coiled‐coil regions in the N‐terminal and C‐terminal regions [53] (Fig. 1). In *T. cruzi*, two different NUP‐1 isoforms of different sizes are apparently expressed from a single gene, however, the functional relevance of this is unexplored [58]. Remarkably, NUP‐1 is seven times larger than mammalian lamins and consequently spans a more extensive volume of the smaller trypanosome nucleus (Fig. 3A). Confocal microscopy suggests that NUP‐1 has a predominantly extended conformation, observed by imaging an N‐terminal tagged NUP‐1 and the central repeats, with resolution between the two signals, suggesting an extended conformation [52].

NUP‐1 localises to the nuclear periphery during interphase, while NUP‐2 localises to the nuclear periphery and nucleoplasm (Fig. 3B) [52, 53, 59]. Both have roles in nuclear integrity as depletion results in nuclear blebbing, abnormal nuclear enlargement with irregular boundaries and aberrant extensions of the NE [52, 53], a common phenotype of mutated lamins in other systems [60]. Furthermore, NUP‐1/NUP‐2 have contacts with the NPC, as evidenced by interactions with TbNup98 and other nucleoporins [59, 61], suggesting that the NPC may facilitate anchoring at the NE, but if these interactions have additional functions besides simple structural support is unknown.

NUP‐1 also influences telomere positioning [52], and both NUP‐1/NUP‐2 regulate gene expression as they participate in silencing VSG genes in the bloodstream form, contributing to monoallelic VSG expression [52, 53]. Furthermore, NUP‐1/NUP‐2 participate in repression of procyclin, the major antigen expressed in the insect stage [52, 53]. Hence, the trypanosoma lamina influences the differentiation and expression of pathogenesis‐related genes. This is also supported by overexpression of NUP‐1 fragments, which leads to the upregulation of important RNA‐binding proteins, including RBP10 [59], a master regulator of differentiation between procyclin and bloodstream forms [62].

*Trypanosoma brucei* presents a highly organised 3D genome architecture and chromosome territories, which influence DNA accessibility and thus, homologous recombination and gene expression [63]. This includes the subtelomeric regions containing VSG genes that are folded into compact domains [63], resulting in a genome‐wide configuration that changes after VSG gene switching [64]. If NUP‐1/NUP‐2 can influence such genome architecture through interactions with chromatin is still unexplored.

The assembly and geometry of the trypanosome lamina is also distinct from mammalian lamins, which form homodimers assembling strictly head‐to‐tail (Fig. 3C) [32]. By contrast NUP‐1 has the flexibility to assemble in a head‐to‐head, tail‐to‐tail or head‐to‐tail manner, forming homophilic or heterophilic interactions between NUP‐1 domains [59] (Fig. 3D), essentially a hub‐and‐spoke architecture. As occurs with mammalian lamins, NUP‐1 and NUP‐2 are codependent in maintaining nuclear shape and integrity, as depletion of either impacts the other, and with the N‐terminal region of NUP‐1 acting as the main anchor point for NUP‐2 [53, 59]. During the cell cycle, distinct NUP‐1 domains locate to specific nuclear subregions during interphase, mitosis and cytokinesis [59], with the C‐terminal region present in the bridge linking the daughter nuclei at late mitosis, while the N‐terminal region is absent from this region [59]. The central repeat region of NUP‐1 is present at the nuclear periphery and nucleoplasm, with this latter location predominant during early mitosis [59]. These observations reflect the flexibility and elastic properties of NUP‐1 and suggest that NUP‐1 domains act to support specific functions and engage with the mitotic machinery. This may reflect the high‐molecular weight of NUP‐1, although in many kinetoplastids the ortholog is considerably smaller than the African trypanosome NUP‐1 [52, 65] (A. Makarov & M. C. Field, unpublished results). NUP‐1 also has multiple potential phosphorylation sites [65, 66] and preliminary data suggests that mutations in those sites lead to aberrations in NUP‐1 dynamics (A. Makarov, L. Koreny & M. C. Field, unpublished data). Moreover, mammalian lamins have additional post‐translational modifications, such as sumoylation [67]; whether such modifications occur and are functionally relevant for NUP‐1/NUP‐2 remains to be explored.

In summary, the extended coiled‐coil proteins NUP‐1 and NUP‐2 constitute major components of the trypanosoma lamina, despite no obvious sequence relationship to mammalian lamins. NUP‐1/NUP‐2 function similarly to Metazoan lamins, supporting roles in maintaining nuclear morphology, interactions with the NPC and chromatin, cell division, and regulation of gene expression, including genes related to development.

### Conclusions and perspectives: nuclear lamina

One of the crucial scaffolds in the cell, the nuclear lamina is an exemplar for molecular evolution and diversity. The identity of the nuclear lamina in many eukaryotic clades is awaiting discovery and characterisation, including many protozoa of medical importance. Interestingly, although lamins, NMCPs and NUP‐1/NUP‐2 systems are different in sequence, the overall functions of the corresponding lamina are highly conserved, acting always as an architectural scaffold of the nuclear structure with a predominant role in chromatin and gene expression regulation. There are many unresolved questions, for example, how do NUP‐1/NUP‐2 influence genome architecture in trypanosomes and regulate expression of VSGs and procyclins? Moreover, as the primary structures of lamin analogues are divergent and taxa‐specific, it remains to be uncovered what, if any, analogues are present in many pathogens, including the malaria parasite and its relatives, *Toxoplasma* and *Cryptosporidium*.

## The nuclear pore complex

**Fig. 4:**
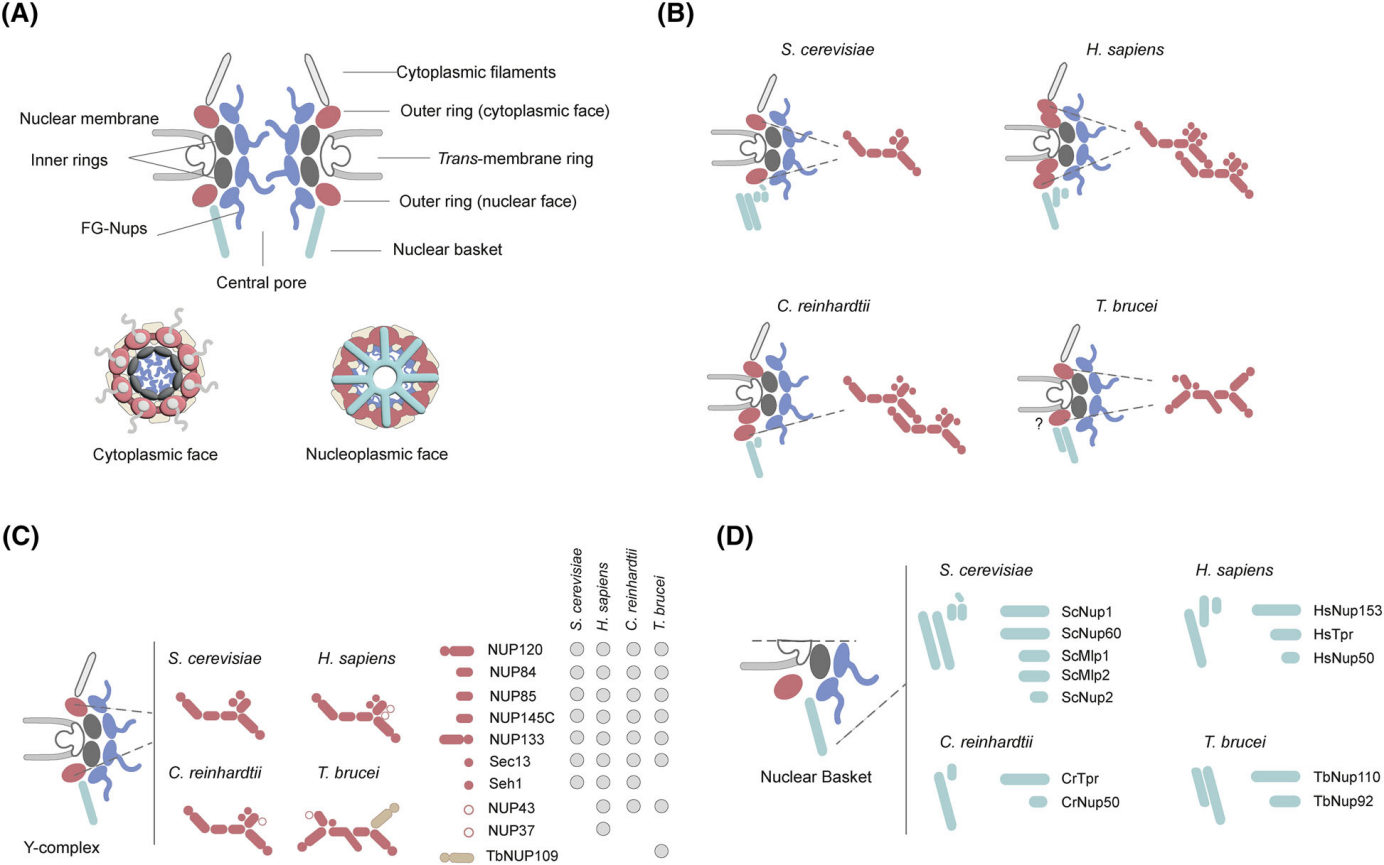
NPC features in eukaryotes. (A) Schematic of the NPC structure showing rings and components. Views from the NE plane, cytoplasm and nucleoplasm are presented. (B) Schematic showing how the outer rings (OR, burgundy) are configured in different organisms. The NPC in *Saccharomyces cerevisiae* has one OR at the cytoplasmic face of the NE and one OR at the nucleoplasmic face. The *Homo sapiens* NPC bears two ORs at each side while the algae *Chlamydomonas reinhardtii* has two ORs at the nucleoplasmic side and only one at the cytoplasmic side. Evidence points that *Trypanosoma brucei* has only two ORs but awaits confirmation. (C) Schematic showing localization of the Y‐complex (burgundy) and comparison of its architecture in four different organisms; *S. cerevisiae*, *H. sapiens*, *C. reinhardtii* and *T. brucei*. Left, homologue subunits are depicted in the same burgundy colour, taxa‐specific subunits in other colours. Right, Coulson plot showing the number and different homologues of the components of the Y‐complex in the mentioned species. (D) Schematic highlighting the different composition of the nuclear basket in organisms in panel C. Figure shows the number and different homologues of the components of the NB in *S. cerevisiae*, humans, *C. reinhardtii* and *T. brucei*.

The NPC is a macromolecular channel allowing bidirectional trafficking between the nucleus and the cytoplasm. The NPC consists of ~ 30 different nucleoporins (Nups) with multiple copies of each, adding up a molecular mass of ~ 50 to 120 MDa in yeasts and mammals, respectively, due to stoichiometry and structural variation [68, 69]. The NPC is built from multiple subcomplexes arranged in rings: (a) cytoplasmic filaments (CF); (b) outer rings (OR), one embedded in the cytoplasmic face of the NE and one nuclear ring at the inner face of the NE; (c) a *trans‐*membrane scaffold ring (MR), an anchoring point for NPC subcomplexes; (d) a central pore, that contains highly mobile phenylalanine‐glycine (FG)‐repeat proteins creating a permeability barrier regulating cargo transport and (e) the nuclear basket (NB) [68, 70] (Fig. 4A). A substantial proportion of the NPC consists of FG‐repeat proteins, intrinsically disordered proteins that interact with cargo and cargo receptors and are located at the central pore, the CF and the NB [68, 70]. One of the hallmarks of NPC is an octagonal cylindrical structure. The NPC has an eight‐fold rotational symmetry around the central transport channel (Fig. 4A). Due to its complex character, we consider the structure and function of the NPC separately. In the NPC structure section, we will refer constantly to architecture in humans and yeast as most structural characterisation comes from studies in these organisms. We will then compare with trypanosomes and other parasites.

### NPC structure

High‐resolution structures of NPCs from mammals, *S. cerevisiae*, *Xenopus laevis*, *D. discoideum* and *Chaetomium thermophilum* are available [71, 72, 73, 74, 75, 76, 77, 78, 79, 80, 81, 82], while the protein components have been identified for *Schizosaccharomyces pombe* [83, 84], *Chlamydomonas reinhardtii* [85], *A. thaliana* [86], *Malus domestica* [87], *Tetrahymena thermophila* [88] and *T. brucei* [61, 89] (Fig. 2). These data have allowed evolutionary reconstruction [23, 90], highlighting that overall morphology, organisation and architecture of NPC subcomplexes are conserved but also demonstrating that lineage‐specific subunit stoichiometry is present within NPCs. Thus, NPC architectural diversity is produced through gains and losses of components together with varying stoichiometry.

The NPC contains two outer rings (ORs), one at the cytoplasmic face and one at the nucleoplasmic face (Fig. 4A). The major building block of the outer ring is the Y‐complex (Fig. 4B) which owes its name to its ‘Y’ shape [72, 74, 79]. In metazoan NPCs (human and *Xenopus*), the cytoplasmic and nuclear outer rings are constituted by two staggered rings of eight Y‐complexes (Fig. 4B). Eight of this Y‐complex doublet forms a ring arrangement, i.e., 32 copies of the complex per NPC [80]. The Y‐complex is a source of great variability in the NPC across the eukaryotes due to two features; (a) the number of Y‐complex copies per NPC and (b) the number of nucleoporins constituting this complex (Fig. 4B,C) [78, 80, 82]. For example, in *S. cerevisiae*, these outer rings are composed by a single ring of eight Y‐complexes, i.e., 16 copies [77] (Fig. 4B). In the green algae *C. reinhardtii*, the cytoplasmic ring contains a single ring of eight Y‐complexes whereas the nucleoplasmic ring consists of 16 copies forming two rings [85] (Fig. 4B). A more divergent arrangement is present in *Schi. pombe*, in which the Y‐complexes are of differing composition between the nuclear and cytoplasmic copies [23, 73, 83, 84] (Fig. 4B). In *T. brucei*, evidence points to one ring of Y‐complex per outer ring (Fig. 4B) [61] but this awaits confirmation. Variability in the Y‐complex is also introduced by distinct component composition, with 6–10 nucleoporins present depending on species (Fig. 4C). In *S. cerevisiae*, there are seven subunits: ScNup84, ScNup85, ScNup120, ScNup133, ScNup145C, ScSec13 and ScSeh1 [73, 77, 91], but in humans nine subunits, including orthologs to yeast components together with two additional subunits, HsNup43 and HsNup37 (Fig. 4C) [79, 80]. In *T. brucei*, the Y‐complex contains eight subunits, seven orthologs to the yeast cohort, *albeit* that trypanosomes lack a Seh1 ortholog, but replace this with an ortholog of HsNup43, TbNup41 (Fig. 4C) [61].

Moreover, the outer rings connect to the nuclear basket (NB), a structure that protrudes from the NPC into the nucleoplasm (Fig. 4A). The NB is an essential platform for RNA export by docking export factors and facilitating transport [68, 70]. Across eukaryotes, the NB is constituted by a different number of subunits, for example, in humans it is constituted by three subunits, HsNup153, HsTpr and HsNup50 [92, 93], while in yeast it is constituted by five subunits, ScNup1, ScNup60, ScMlp1/2 and ScNup2 [73, 77], and in algae NPC basket contains only two subunits [85] (Fig. 4D). As some of the Nups present in these platforms are dynamic, exact functions remain unclear. For example, in mammalian cells it is not known if HsNup153 is essential for Tpr attachment to the NPC as evidence is contradictory [94]. Moreover, apart from RNA export, the basket Nups have been implicated in chromatin remodelling and control of gene expression [70]. Regarding RNA export, in eukaryotes this process is helped by different complexes; Mex67‐Mtr2, TREX and TREX‐2 [23, 95, 96], which interact with NPC components to coordinate the export. The NB has also been suggested to act as a quality control point for RNA export, as it distinguishes between normal and aberrant mRNAs, although the mechanisms for this are unclear [23, 97, 98].

In trypanosomes, the NB consists of at least two proteins, TbNup92 and TbNup110 (Fig. 4D) which are Kinetoplastid restricted and divergent from NB components in humans and yeast [61, 99]. Additional proteins that are likely NB components have also been identified by proteomics, and which also bear predominantly coiled‐coil structures (E. R. Butterfield, S. O. Obado, S. R. Scutts, W. Zhang, B. T. Chait, M. P. Rout & M. C. Field, unpublished results). Export factors are also divergent across taxa and classical mammalian complexes such as TREX and TREX‐2, regulating mRNA splicing, export and aiding in translocation of mRNPs to the NPC, are essentially absent from trypanosomes [61, 100]. Again, this suggests that the complexes interacting with the NPC evolved distinctly in trypanosomes. A remarkable example of this is the recent characterisation of Mex67 paralogues in trypanosomes [101, 102]. Mex67‐Mtr2 is the main mature mRNP carrier in yeast (Nxf1‐Nxt1 complex in humans), essential for translocation of the mRNP to the FG‐Nups in the NPC [103]. *T. brucei* is the first protozoa identified to have multiple paralogues of Mex67; TbMex67, TbMex67b and TbMex67L, all encoded by different genes [102, 104]. TbMex67 and TbMex67b localise to the nucleolus and the NPC and interact with multiple NPC components and are required for mRNA export. By contrast TbMex67L localises exclusively to the nucleolus and instead interacts with ribosomal biogenesis factors and ribosomal components [102]. Furthermore, paralogs Mex67 and Mex67b interact with the small GTPase Ran, suggesting a Ran‐dependent mechanism of RNA export [61, 102, 104]. This represents another hallmark of trypanosomes, as in animals, fungi and plants, the bulk mRNA export occurs in a Ran‐independent manner [61, 102].

The NPC outer rings also connect to the cytoplasmic filaments (CFs), that establish transport directionality and provide docking sites for transport factors contributing to the mRNA export process (Fig. 4A) [72]. Residing at the OR at the cytoplasmic side, they are mainly constituted by the yeast Nup82 complex [73, 91]. This complex recruits the RNA helicase Dbp5 and associated factors such as Gle1 and Gle2 [105] that release the mRNA to the cytoplasm. In yeast, the Nup82 complex is constituted by three proteins, ScNup82, ScNup159 and ScNup42, while in humans it is constituted by HsNup214, HsNup88 and HsNup62 [80]. In *T. brucei*, the equivalent Nup76 complex is constituted of TbNup76, TbNup140 and TbNup149 [61]. Of these, only TbNup76 shows homology to ScNup82, while the other two subunits are apparently taxon specific [23, 61]. Regarding associated factors, only Gle2 has been found in trypanosomes [23, 61], with the possibility that taxa‐specific proteins interacting with the CF for the release of the mRNAs from the NPC are yet to be discovered.

**Fig. 5:**
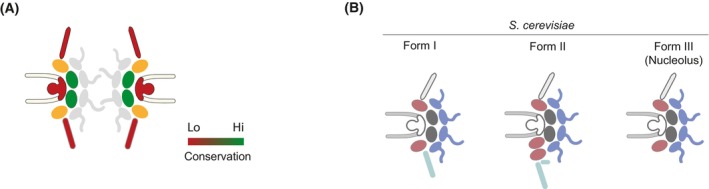
Heterogeneity in the NPC. (A) Levels of conservation along the NPC among eukaryotes. Green, high conservation, red low conservation. (B) Structural variants of the NPC present in the same cell detected in *Saccharomyces cerevisiae* [116]; Form I corresponds to the canonical NPC, Form II includes an extra OR in the nucleoplasmic side and Form III lacks the NB and is detected in proximity to the nucleolus.

The inner ring is the structural core and most conserved NPC element (Figs 4A and 5A). IRs extend from the NE membrane to the central channel, shaping and stabilising the NE and providing an anchor for remaining NPC core components and FG Nups. There are three subcomplexes constituting the IR extensively studied in yeast; the ScNup49‐ScNup57‐ScNsp1 heterotrimer and two heterodimers ScNup188‐ScNup192 and ScNup157‐ScNup170 [106]. ScNup49‐ScNup57‐ScNsp1 constitute part of the central transport channel and form a diffusion barrier with their disordered FG repeats [106]. These interact with ScNic96, an evolutionary highly conserved protein that anchors channel Nups with the ScNup192‐ScNup188 subcomplex (both α‐solenoid). A deeper layer of the IR, interacting with the membrane ring and ScNic96, is the β‐α‐structured subcomplex ScNup157‐ScNup170. A difference between organisms is the presence of extra copies in humans of Nup155 (ortholog of ScNup157/ScNup170) forming additional pillar structures in the vertebrate NPC [77, 79] and present only in the nuclear IR in the algae *C. reinhardtii* [85]. *T. brucei* presents orthologs for all of these proteins, namely, TbNup225, TbNup181 (α‐solenoids), TbNup119, TbNup144 (β‐α‐structured), TbNup96 (ortholog of ScNic96) and FG‐Nups, TbNup53a, TbNup53b and TbNup62 (orthologs of ScNup57, 47 and Nsp1, respectively) [23, 61], showing again the conservation of these NPC elements across eukaryotes.

By contrast, the *trans*‐membrane ring has a diverse arrangement, with little sequence conservation across eukaryotes [23, 91]. The MR is composed of pore membrane proteins (POMs), four identified in humans and yeast, but only Ndc1 and POM33 have orthologs between species, with the remaining POMs apparently taxa‐specific [23]. Moreover, the exact functions of individual POMs remain elusive, beyond contributions to stabilising connections between the NE and the IR [80]. So far, no orthologs for POMs have been detected in trypanosomes [23, 61] or other pathogens.

Apart from Kinetoplastid parasites, characterisation of NPC components in several medically important protozoa is in progress, including the Alveolata *Toxoplasma gondii* [107], *Plasmodium berghei* and *Plasmodium falciparum* [108, 109] (Fig. 2), and emphasise divergence as several Nups are significantly larger than homologues in animals and fungi. For example, in *P. falciparum*, one of the more virulent species causing malaria, nucleoporin PfSec13 is 90 kDa *versus* animal/fungal Sec13 at 35 kDa. This configuration has been generated by fusion at the C‐terminal region with a region similar to yeast Nup145C [110]. In *To. gondii*, TgNup302 is also significantly larger than the yeast homologue Nup100 (302 kDa *versus* 100 kDa, respectively) and bears an autocatalytic domain that generates two polypeptides of 150 and 170 kDa respectively [107]. These observations suggest that Apicomplexan NPCs assemble by distinct pathways from animals and fungi. Interestingly, the localisation and number of NPCs varies considerably between developmental stages in *Plasmodium*, ranging from three to ~ 60 NPC per nucleus in erythrocytic stages (ring, trophozoite and schizonts) [111]. This may reflect the transcriptional state of the cell and/or metabolic activity as it clearly does in metazoan cells [112, 113].

The evolution of the NPC is associated with the protocoatomer hypothesis, which proposes that a huge number of membrane‐associated complexes, including the NPC, have a common evolutionary origin as they share deep architectural similarities [23, 54]. Protocoatomers are membrane‐deforming proteins constituted of β‐propeller and α‐solenoid domains, whose secondary structure (but not sequence) is highly retained between many subcellular compartments. There are two distinct subfamilies; Type I, featuring an N‐terminal β‐propeller followed by a continuous α‐solenoid and Type II, constituted by an α‐solenoid but with a break in the α‐solenoid [23, 54]. NPCs contain proteins from both subfamilies, suggesting NPC architecture may have been established late during eukaryogenesis, but also implying that a basic nucleocytoplasmic transport system is ancient and was established pre‐LECA [114]. Bioinformatic analysis of NPC subunits show that these are not conserved in sequence among eukaryotes but that the proteins retain recognisable conserved domains and secondary structures. A minor proportion of Nups are lineage specific, e.g., TbNup140 and TbNup149 in *T. brucei*, a potential indication of functional diversification [19, 23, 54].

All these studies and observations indicate a common structural design for the NPC across eukaryotes [91, 100, 114], represented in Fig. 4A, but that different taxa have adaptations to this generalised plan. As mentioned, the eukaryotic NPC architectural diversity is produced through gains and losses of components together with varying stoichiometry. In terms of conservation, the IR is the most conserved NPC substructure (Fig. 5A) while subunits in the basket and cytoplasmic filaments are less conserved. A rational for divergent proteins in the trypanosome NB and CF is that they have adapted to interact codependently with an also divergent mRNA export pathway (Fig. 5A). An alternate view, expressed by one of us recently (Mark C. Field in [115]), is that there are so many differences within RNA processing pathways and NPC composition between trypanosomes and other lineages that this may reflect a more fundamental distinction, and that trypanosomes could be examples of an earlier eukaryotic state [115]; however, the validity of either argument remains unestablished. Apart from variations in the NPC architecture among eukaryotes, variations in composition have been detected among tissues and even in the same cell.

### Nuclear pore heterogeneity in the same cell

Studies in yeast have highlighted that the NPC does not have a uniform structure, but rather it can vary even within a single cell, as NPCs of at least three distinct compositions coexist in the same cell at the same time in *S. cerevisiae* [116, 117, 118]. Form I is predominant and has the canonical arrangement of two outer rings (OR). Form II contains three ORs, two at the nucleoplasmic side and with enrichment of nucleoporin Nup188 in the nuclear basket (normally in the inner ring). Form III contains the canonical two ORs but lacks nuclear basket subunits and is abundant (but not exclusive) in NPCs proximal to the nucleolus (Fig. 5B). So far, Form III are the only of these forms functionally characterised in *S. cerevisiae* [117, 119], highlighting that basket‐less NPCs are the default assembly state and that baskets only assemble in an mRNA/mRNA‐dependent manner [117, 119]. Active RNA Polymerase II transcription and the presence of mature, processed mRNAs are requisites for the assembly of the basket Nups [119]. Importantly, the basket serves as a quality control platform to ensure that non‐processed mRNAs stay in the nucleus [119]. The proximity of basket‐less NPCs to the nucleolus was suggestive of selective transport of ribosomal subunits through these NPCs for ribosomal production, however, it was later shown that this transport can occur through nuclear pores located far from the nucleolus [120]. Thus, the purpose of basket‐less nuclear pores in proximity to the nucleolus is still to be determined. Moreover, it is still unclear how Forms I and II compositions may impact function. These observations highlight that the NPC is heterogeneous even in a single cell; whether there are different NPC compositions in parasites engaging in different cell functions is unknown.

### Nuclear pore heterogeneity among tissues

In mammals and plants, a tissue‐specific expression of NPC components has been described [121, 122], as protein and mRNA levels vary across cell types of healthy and pathological tissues [121]. Interestingly, this tissue‐dependent expression is linked to cell differentiation, tissue development and function [121]. Protein and mRNA expression profiles in human tissue culture cells indicate cell type‐specific changes to expression of certain NPC components, i.e., cell‐specific changes have been observed for HsNup50, Tpr (nuclear basket), HsAladin, HsNup214 (cytoplasmic filaments), HsGp210, HsPOM121 (membrane ring) and HsNup37 (inner ring) [121]. Changes to Nup expression across tissues may indicate variation in NPC assembly, and hence is suggestive of the existence of specialised nuclear pores with cell type‐specific functions [121]. Moreover, these differences in expression, together with loss‐of‐function mutations in Nup genes, can cause tissue‐specific phenotypes [121]. For example, in humans, HsNup155 is highly expressed in the heart and when mutated, leads to cardiac disease [123]. How these differences in stoichiometry NPC subunits impact normal and aberrant functions is an important area of current investigation.

### NPC functions

The main function of the NPC is nucleocytoplasmic trafficking but the structure supports roles in additional activities, such as modulation of chromatin, regulation of gene expression (gene activation or silencing; epigenetic gene regulation; regulation of transcription and mRNA export) and transcriptional activity (assembly of transcriptional complexes). Moreover, the NPC subunits can participate in transcriptional memory, i.e., the cellular ability to enhance transcriptional response upon an external cue, which can be maintained epigenetically [124]. As discussed, the NPC represents a docking site for RNA export complexes through the NE and CF. Interestingly, the NPC influences development and differentiation in *Drosophila* as well as hormonal and immunological signalling in plants [124]. Moreover, consistent with many functions occurring in the nucleoplasm, some Nups shuttle between the NPC and the nuclear interior. In several organisms, including yeasts, humans, *Drosophila* and trypanosomes, soluble dynamic Nups bind transcriptionally active genes and recruit them to the nuclear periphery to regulate gene expression [68, 70].

In recent years, different diseases have been linked to mutations in the genes of Nups, disorders that have been termed ‘nucleoporopathies’ [72]. These diseases can also arise through alterations in the NPC composition, in subunit levels (sequestration, aggregation or overexpression) or in nucleocytoplasmic transport [72]. Such alterations lead to a range of pathologies, including neurodegenerative diseases (e.g., dementia, Huntington's, Alzheimer, Parkinson's) [125], autoimmune disorders (Systemic lupus erythematosis, rheumatic disease), cancer [126], renal [127, 128] and ovary dysfunction [129, 130].

Trypanosome Nups perform conserved functions observed in higher eukaryotes, as they participate in lamina anchoring (TbNup98) [52, 59], tRNA trafficking (TbNup62 and TbNup53a) [131] and mRNA processing through interactions with the *trans*‐splicing machinery [132]. Similar functions are being unravelled for other parasites, as in *To. gondii*, in which TgNup302 participates in trafficking of the ribosomal RNAs and also interacts with histone modification enzymes, suggest a role in gene regulation [107]. Trypanosome Nups also actively participate during mitosis, as TbNup92 (located in the NB) associates with the spindle pole body during cell division to ensure correct chromosome segregation [99] while TbNup144 plays a role in mitosis checkpoints [131]. These roles reflect conservation of Nup functions in cell division across eukaryotes. As happens in mammals, some trypanosome Nups, namely, TbNup53b and TbNup92, are dynamic and shuttle between the NPC and nucleoplasm to fulfil roles in the nuclear interior to interact with the *trans*‐splicing machinery and to contribute to chromatid segregation, respectively [99, 132].

The dynamics of NPC and NE components during nuclear division remain essentially uncharacterised in parasites. Although open and close mitosis seem distinct, the mechanisms underpinning disassembly of the NPC are highly similar and occur in a synchronised manner. In *Aspergillus nidulans* and *Schi. pombe*, during anaphase the intact daughter nuclei are only connected by a narrow bridge from where NPC components gradually disassemble; peripheral NB Nups detach first from the NPC, followed by central scaffold Nups and finally, POMs [133, 134, 135, 136]. In *T. brucei*, evidence points towards a similar mechanism, where NB Nups (TbNup92) [99] are first lost from the mitotic bridge, followed by structural Nups (TbNup158, TbNup53b) [89, 132], however, the exact mechanisms for NPC disassembly during mitosis in trypanosomes remain to be explored in detail.

Nups in parasites can openly contribute to regulating expression and interestingly, do so with genes related to developmental stage or pathogenesis. In *T. brucei*, TbNup53b contributes to regulation of expression of procyclins, normally expressed only in the procyclic stage, but depletion of TbNup53b results in the expression of procyclins in the bloodstream form [132]. How Nups in trypanosomes contribute to regulate expression is unknown, but in other eukaryotes is finely orchestrated by Nups in collaboration with the nuclear lamina, epigenetic (histone) marks and RNA processing factors, for example, plants [137], *S. cerevisiae* [138], *Schi. pombe* [139, 140] and *Drosophila* [141, 142]. Less explored are the roles of NPC components in virulence and infection, e.g., *Leishmania*, an intracellular protozoan parasite, uses the metalloprotease GP63 to degrade host Nups and nuclear receptors, altering the composition of the infected host cell [143, 144], while *Theileria annulata*, a bovine intracellular pathogen, recruits NPC components of the host cell to the surface to form porous structures of unknown function [145].

### Conclusions and perspectives: the NPC

One of the largest macromolecule complexes in the cell, the NPC is a superb exemplar of molecular evolution, diversity and adaption. Each lineage possesses an NPC which has evolved and adapted to meet specific biological constraints. Although the NPC core is mostly conserved, remaining NPC components have adapted to interact with transcription, mRNA processing and mRNA export machineries that are frequently highly specialised between lineages. Nevertheless, there are many unsolved questions about the NPC components in parasites, for example, about their precise role in gene expression and transcription regulation or whether and how those components may, or may not, influence virulence or development. Moreover, while the overall functions of the NPC are highly conserved, there are significant differences in the mechanisms by which these are supported, providing a complex tangle of convergent and divergent evolutionary pathways.
